# Utilization of psychotropic medications in children with FASD: a retrospective review

**DOI:** 10.1186/s12887-021-02986-5

**Published:** 2021-11-16

**Authors:** Michael-Roy R. Durr, Susan Petryk, Mansfield Mela, Andrea DesRoches, Mackenzie Wekerle, Sanjida Newaz

**Affiliations:** 1 College of Medicine, University of Saskatchewan, Saskatoon, Saskatchewan Canada; 2 Research Department, Saskatchewan Health Authority, Saskatoon, Canada

**Keywords:** Fetal Alcohol Spectrum Disorder, Prenatal Alcohol Exposure, Medication Algorithm

## Abstract

**Background:**

Fetal Alcohol Spectrum Disorder (FASD) is a neurodevelopmental condition resulting from pre-natal alcohol exposure. In Canada, an estimated 1.4-4% of newborns are affected by FASD. FASD is often associated with behavioural comorbidities and many individuals require psychotropic medication. However, to date there are no FASD specific guidelines for prescribing medication. Recently, Mela and colleagues described four behavioural symptom clusters commonly seen in FASD with suggested pharmacologic treatment for each cluster within an algorithm. The primary objective was to compare the proposed treatment algorithm retrospectively to actual treatment in a real-world FASD pediatric practice. The secondary objective was to refine the description of symptom clusters which will be targeted with treatment.

**Methods:**

We collected the diagnostic and medication history from all patient visits of a Regina Developmental Pediatrician who specializes in FASD diagnosis and medication treatment. Three hundred fifty-four FASD patients were identified between 2005 to 2020. The medications that would be predicted from the algorithm were compared to the real-world historical data. A positive case was defined as all algorithm-predicted medications matching the historical data; a negative case had one or more medications failing to match.

**Results:**

Of the 354 patients, 36 were removed for insufficient information. Of the remaining 318 cases, 172 (54.1%) were positive compared to 146 (45.9%) negatives. In single prescription cases (*n*=147), the incidence of positives was 67.3%; in multi-prescriptions (*n*=72) it was 27.8%; and in cases where no prescription was needed (*n*=99), the positive incidence was 53.5%.

**Conclusions:**

The prescription algorithm is promising but requires further refinement to accommodate the range of presentations in children with FASD. With respect to unclassified symptoms, we propose the following: sleep onset difficulty as *hyperarousal*; gender dysphoria and obsessive compulsive disorder as *cognitive inflexibility*; grief as *emotional regulation*; and autism spectrum disorder as *hyperactive/neurocognitive*.

## Background

FASD is a complex brain condition associated with prenatal alcohol exposure (PAE) affecting cognitive, neurological, social and interpersonal functions leading to lifelong disability. Neurodevelopmental disorder associated with PAE (ND-PAE) is recognised in the DSM-5. In Canada, an estimated 1.4-4% of babies are born with ND-PAE/FASD [1]. The comorbidities associated with ND-PAE/FASD are wide ranging and can affect multiple areas of an individual’s functioning, including their cognition, behaviour, mood, and general wellbeing. Many children with FASD require psychopharmacological treatments for comorbid emotional and behaviour problems.

While clear diagnostic thresholds and guidelines exist for ND-PAE/FASD [2], guidelines for treatment of the condition are still emerging. Currently, there are well agreed upon guidelines for psychosocial management in children with ND-PAE/FASD; the approach is largely targeted at the individual’s personal relationships, as well as their symptoms specific to their particular neurodevelopmental deficits [3]. Most recommended psychosocial interventions have been subjected to rigorous randomized double blind control studies providing the evidential basis [4]. With respect to psychotropic medication interventions, the research is limited, and the findings lack consensus [5, 6]. The challenge herein is the result of the multiple comorbidities associated with ND-PAE/FASD: medical management risks overmedicating individuals and producing multiple adverse effects [7, 8]. Furthermore, many cases are underdiagnosed and have variance of symptom severity among patients and throughout an individual’s life [8–11]. Evidently, the challenges inherent to prescribing for ND-PAE/FASD are significant, especially for clinicians with limited exposure to the disorder.

**Fig. 1:**
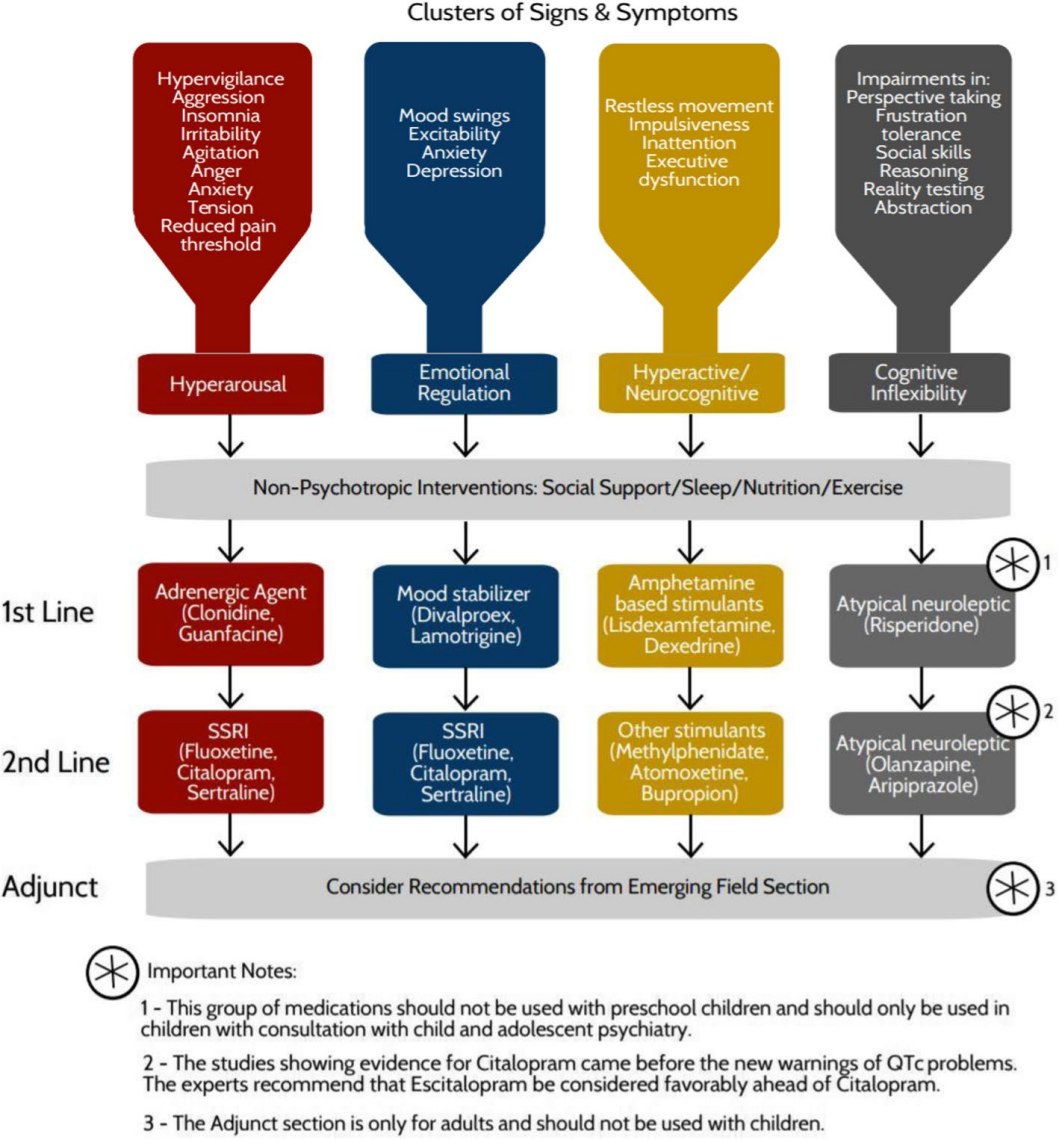
Algorithm approach to FASD symptomatic clusters (used with permission) [14]

The literature concerning medical intervention in ND-PAE/FASD is centred around stimulants, mood stabilisers, and medicines influencing dopamine, serotonin, and norepinephrine receptors [7, 12]. Early research in this area has shown promising treatment algorithms for disorders related to ND-PAE/FASD, namely, attention deficit hyperactivity disorder (ADHD), post-traumatic stress disorder (PTSD), depression, and anxiety [13]. The need for a treatment algorithm for the entirety of ND-PAE/FASD is well known and has been advocated for by the Canada FASD Research Network and their Family Advisor Committee. As a result of this call to action, a multidisciplinary panel of experts was formed and developed an algorithm for the psychotropic medical management of individuals with ND-PAE/FASD. From their work, four clusters representing ND-PAE/FASD was outlined and a treatment for each cluster was proposed (Fig. 1.) [14].

The four clusters are as follows [14]:

1. Hyperarousal: deficits to the brain stem, midbrain, and limbic areas, resulting in mood dysregulation and impaired executive functioning. First line treatment: adrenergic agents; second line: SSRI.
2. Emotional Regulation: deficits to the hippocampus, amygdala, HPA axis, and prefrontal cortex, resulting in emotional dysregulation and abnormal social behaviours. First line treatment: mood stabilisers; second line: SSRI.
3. Hyperactive/Neurocognitive: negative deficits to the dopaminergic and noradrenergic pathways, resulting in hyperactive behaviour. First line treatment: amphetamine stimulants; second line: other stimulants.
4. Cognitive Inflexibility: deficits to the frontal lobe, resulting in poor adaption function. First line therapy: atypical neuroleptics; second line: atypical neuroleptics.

Currently, no research has been conducted to evaluate the accuracy of the proposed treatment algorithm.

## Methods

We sought to apply the algorithm in a retrospective review to evaluate the validity, as well as to provide recommendations on how to refine the algorithm for management of children with ND-PAE/FASD. The primary objective was to compare the proposed treatment algorithm retrospectively to actual treatment in a real-world FASD pediatric practice. The secondary objective was to refine the description of symptom clusters which will be targeted with treatment.

The complete patient visit history of a Regina-based Developmental Pediatrician were reviewed to identify all ND-PAE/FASD visits (initial and follow up) between 2005 and 2020. This pediatrician services the entirety of the FASD population in southern Saskatchewan. All ND-PAE/FASD symptoms and psychotropic medications used were compiled for the 354 patients. These patients were assumed to have had good outcomes (well-tolerated medications and controlled symptoms) due to their long history of management under this single pediatrician. We created a master list and classified all presenting symptoms across all patients to align with the clusters defined in the algorithm (see Table 1). Some patients had symptoms that could not yet be classified or had incomplete records and were excluded (*n*=36). There were no other exclusion criteria. Using the master list of symptoms, a patient’s FASD cluster(s) was inferred based on their symptoms across all visits, and their predicted psychotropic medications as per the algorithm was compared to their historical or actual prescriptions. The historical prescriptions were assumed to have good outcomes based on long term follow-up and stabilization being a criterion for discharge from the specialist back to primary care. A “positive case” for the algorithm was defined as all algorithm predicted medications matching the actual historical data. Likewise, a “negative case” had one or more medications failing to match, i.e., the algorithm missed a prescription or prescribed when not needed. Finally, the incidence of positive cases was noted to evaluate the accuracy of the algorithm. To evaluate the strength of the algorithm, Cohen’s Kappa statistic [15] was used. Some patients had symptoms that could belong to more than one cluster than one cluster (e.g. insomnia), such cases were first evaluated to confirm that the medication used in practice was congruent with either of the two potential clusters. Then, the patient’s other symptoms were considered to decide if the case was single or multi-cluster: for instance, a patient with trauma, adverse childhood experiences, and insomnia was classified as only hyperarousal; whereas a patient with trauma, depression, and insomnia was classified as multi-cluster of both hyperarousal and affect regulation. Multi-cluster symptoms like insomnia did not occur in isolation for the patient population. As such, if a multi-cluster symptom like insomnia was noted with a cluster other than hyperarousal/affect regulation, the remainder of the patient’s symptoms were used to decide the best cluster.

**Table 1:** Classification of charted symptoms into FASD clusters according to the algorithm

Hyperarousal	Affect regulation	Hyperactivity	Cognitive inflexibility	Unclassified	
PTSD	Depression	ADHD	ODD	Substance use disorder	Psychosocial disorder
ACEs	Anxiety disorder	ID	Disruptive behavioural disorder	Apraxia	Stutter
Attachment*	Anger*	Borderline IQ	Conduct disorder	ASD	Neglect
RAD	Adjustment disorder	GDD	Perseverative disorder	ABI	Suicidal
Trauma	Emotional dysregulation	Inattentive	Paranoia	SLI	Porn addiction
Insomnia*	Behavioural aggression*		Behavioural defiance	Gender dysphoria	Language delay
Agitation*	Panic disorder*		Running away	Grief	Sexual abuse
Irritability*	Low mood		Executive function impairment	Hair pulling disorder	Memory impaired
	Phobias		Compulsive behaviour	Sleep onset	Tics
	MDD		Conduct problems	Panic attack	Emergent homicidal/suicidal
	EB dysregulation			Abuse	Behavioural instability
				Pseudo seizures	Receptive language delay

Abbreviations: *PTSD* post-traumatic stress disorder, *ACEs* adverse childhood events, *RAD* reactive attachment disorder, *MDD* major depressive disorder, *EB dysregulation* emotional and behavioural dysregulation, *ADHD* attention deficit hyperactivity disorder, *ID* intellectual disability, *GDD* global developmental delay, *ODD* oppositional defiant disorder, *ASD* autism spectrum disorder, *ABI* acquired brain injury, *SLI* specific language impairment

^*^ Indicates a symptom that can be classified as hyperarousal and/or affect regulation

It is also worth noting that we considered evaluating the algorithm by using the pediatrician’s prescription to then derive the FASD cluster(s) as per the algorithm and compare the patient’s symptoms to that of the cluster. We opted for the former approach of using symptoms to determine FASD cluster to then compare management, as this approach is most in line with how the algorithm would be used in real-world practice.

To limit potential bias, the first author (MD) was the sole researcher responsible for extrapolation of data. The second author, who was the practicing pediatrician (SP), was the sole person involved in the real-life symptom management and prescription. The patient symptoms/diagnoses that they (SP) noted were made using clinical history and/or clinical questionnaires as appropriate. These symptoms/diagnoses were then extrapolated from the patients’ paper charts for each visit. The third author (MM) was the sole person responsible for the symptom definitions of the algorithm (Table 1), though the algorithm itself was made by a large group of researchers for which MM was the lead author. To avoid potential biasing of the data/results, MM was given a list of all the 58 symptoms that were listed at least once to then classify them according to the FASD clusters. MM was not told the frequency of these symptoms nor were they told with what symptoms they typically occurred with. Afterwards, MD independently used this list to stratify the patients to FASD clusters and perform the data analysis.

## Results

Of the 354 patients, 213 (60%) were male patients, and 141 (40%) were female. The age range at time of first visit was 3 to 20 years of age, with a mean and median age of 11 years, and a standard deviation of 3 years (Table 2). Of the total patients, 36 were removed for insufficient information. Of the remaining 318 cases, there were 172 (54.1%) instances where the pediatrician’s management matched the recommended psychotropic on the algorithm (positive case). In contrast, 146 patients (45.9%) did not have agreement between the pediatrician’s approach and the algorithm (negatives). This level of positive cases reflects moderate accuracy according to Cohen’s Kappa statistic.

**Table 2:** Age and sex of FASD patients

Age (years)		Sex	
Range	3 – 20	Male	213 (60%)
Mean	11	Female	141 (40%)
Median	11		
Standard deviation	3		

By examining the unclassified symptoms and the management approach of the pediatrician for these cases, we propose the following cluster stratifications: sleep onset difficulty as *hyperarousal*; gender dysphoria, and obsessive compulsive disorder as *cognitive inflexibility*; grief as *emotional regulation*; and autism spectrum disorder as *hyperactive/neurocognitive* (Table 3).

**Table 3:** Proposed clusters for unclassified symptoms

Symptom	Number of cases	Proposed cluster	Number of cases where medication matches hypothetical cluster
Gender dysphoria	2	Cognitive inflexibility	2 (100%)
Substance use disorder	5	Cognitive inflexibility	3 (60%)
Sleep onset	9	Hyperarousal	5 (56%)
OCD	3	Cognitive inflexibility	3 (100%)
ASD	2	Hyperactive/Neurocognitive	2 (100%)

In the pediatrician’s historical data, there were one of three outcomes: a single prescription was needed, a multi-prescription was needed, or no medications were needed. We further examined the incidence of positive and negative cases through these specific subtypes (Table 4). In single prescription cases (*n*=147), the incidence of positives was 67.3%; in multi-prescriptions (*n*=72) it was 27.8%; and in cases where no prescription was needed (*n*=99), the positive incidence was 53.5%. The respective accuracies according to Cohen’s Kappa statistic are strong (single prescription), weak (multi-prescription), and moderate (no prescription needed).

**Table 4:** Accuracy of algorithm in different case types

Case Kind	Incidence	Positive cases	Percent positive	Negative case	Percent negative
Single prescription	147	99	67.3	48	32.7
Multiple prescription	72	20	27.8	52	72.2
No prescription	99	53	53.5	46	46.5

## Discussion

The proposed ND-PAE/FASD psychotropic algorithm predicted the paediatrician’s psychotropic medication choice for 54.1% of the clinic population, which suggests moderate accuracy according to Cohen’s Kappa statistic. While seemingly low, this incidence is promising for the future application of the algorithm. It should be noted that this algorithm’s intended use is alongside a clinician’s judgement. In this study, the prescription was completed without initial clinician guidance (a variance from the intended use of the algorithm) and being retrospective, this data collection missed the clinical context. In turn, the algorithm cannot account for changes in prescription due to side effects or ineffectiveness. Since the very purpose of the algorithm is to reduce polypharmacy, as expected, the algorithm failed to predict cases requiring polypharmacy (predicting single prescription cases (67.3%) vs multi prescription cases (27.8%)). This difference could be due to lack of clinician guidance or could be due to a limitation of the algorithm in the clinically complex cases.

Where the algorithm performed best was in single FASD cluster scenarios (67.3%), while it struggled in multi-prescription (27.8%) and no prescription cases (53.3%). We propose that the group differences are largely informed by the absence of clinician guidance. For no prescription cases, the algorithm recognises that non-psychotropic interventions are always first line for every cluster, however the algorithm cannot apply and evaluate such treatment by itself. The struggle with multi-prescription cases may further be informed by the variance of symptom presentation. A great challenge for treating FASD lies in the neurodevelopmental and symptom differences *between* individuals as well as *within* an individual throughout the developmental stages over a lifetime [8–11]. Moreover, the pediatric history is largely obtained second hand from a care giver. Future studies should examine if the accuracy of the algorithm increases with a pediatric patient’s age when individuals can give a self-report of symptoms. This may then suggest improved algorithm prediction if the patient can communicate on their own behalf.

We do recognise that the historical records are not infallible. The potential for misclassification of symptoms must be noted for FASD clusters with close overlap; in particular, the hyperarousal cluster with emotional dysregulation, as well as hyperactivity/neurocognitive cluster overlap with cognitive inflexibility. This overlap could inform some of the negative algorithm predictions. For instance, symptoms like hyperactivity and impulsivity can readily be misidentified as a behavioural disorder representing cognitive inflexibility, which in turn results in a negative prediction from the algorithm. As well, some researchers suggest ADHD may be over-diagnosed in children [16, 17]. This may explain why the algorithm over prescribed medication in some cases as the initial patient charts may have been influenced accordingly.

Some general patterns emerged from the algorithm. The algorithm often correctly predicted management for ND-PAE/FASD patients of the hyperactivity/neurocognitive cluster. We hypothesise this to be because externalizing behaviours are easier to identify by parents/caregivers. Within the cluster of hyperactivity/neurocognitive, the algorithm predicted medication for all cases of intellectual disability when this study data showed only half actually needed treatment (48%). This emphasises viewing the algorithm as merely a tool to be used alongside clinical judgement. Furthermore, the algorithm was unable to predict when some patients required more than one medication of a single class to treat their FASD cluster. We suggest that this is due to the spectrum of disease severity, which has already been noted in the literature [6].

With respect to cases that were removed for unclassified symptoms, the only cases removed were those where the symptom cluster could not be classified. If the patient had algorithm-classified symptoms amongst its unclassified ones but got a *different* medication than would be predicted by the algorithm, they were categorised as a “negative” case. Only cases where a conclusion could not readily be made were removed as unclassified. By looking at the medication prescribed by the physician in the cases that were removed, we inserted the symptom into the cluster most associated with that medication. Using this method, we propose an adjustment of the clusters as follows: sleep onset difficulty belongs in the hyperarousal cluster; gender dysphoria and obsessive compulsive disorder belong in the cognitive inflexibility cluster; grief belongs in the emotional regulation cluster; and autism spectrum disorder (ASD) belongs in the hyperactive/neurocognitive cluster (Table 3). Given the complex clinical presentation and the overlying executive dysfunction [18, 19], we would suggest the classification of ASD as hyperactive/neurocognitive. Future work should re-evaluate these suggested classifications, especially given our small unclassifiable sample size.

What reasons could explain why the algorithm did not predict the actual medication given in almost half of the cases? A reverse application of the algorithm upon actual clinical practise has some limitations. Judgement and guidance by the treating physician are needed to determine actual symptom severity, presence of impairment from these symptoms, relative impact of multiple symptoms which occur comorbidly, the mitigating effects of adjunct social supports and lifestyle adjustments (adequate sleep, and proper nutrition and exercise) and then, the final determination of whether or not any medication is warranted. Other factors affect choice, including parental preferences, priorities and medication affordability, and insurance coverage.

The results of this study are informed by one pediatrician’s treatment and impressions which affects the generalizability. Future work is needed for a matched comparison population with baseline-outcome findings. As the FASD algorithm research is still in infancy, we felt it best to utilise the whole sample size for a retrospective review.

## Conclusion

The present medication algorithm is promising but requires further flexibility, refinement, and validity testing to accommodate the range of presentations in children and the variance therein. The algorithm is especially strong when predicting FASD single-cluster cases, but the need for physician guidance is evident when prescribing in more complex multi-cluster cases. For those as yet unclassified symptoms, we have provided some logical recommendations. Next steps could include more validity studies like this one, but across more age groups including adults. Ultimately, the algorithm requires a large prospective randomised controlled trial amongst several physicians to test its actual utility. Continued prospective analysis of the emotional behavioural challenges in children and youth with FASD is recommended to further refine the validity of the symptom clusters.

A psychotropic medication algorithm for use in FASD is undoubtedly of great interest to clinicians and to families. Individuals with FASD are already particularly vulnerable and a medication algorithm is greatly needed to guide less experienced physicians who provide medical care. An algorithm could increase physician confidence to make informed medication choices, to prevent over-medication and ultimately lead to the most appropriate psychotropic medication treatment for individuals with FASD.

## Data Availability

The datasets generated and analysed during the current study are not publicly available due to the sensitive patient information contained within. The data generated for analysis is included in this published article. The datasets generated and/or analysed are available from the corresponding author on reasonable request with approval from the local ethics board.

## Abbreviations

FASD: Fetal alcohol spectrum disorder
ND-PAE: Neurodevelopmental prenatal alcohol exposure
PTSD: Post-traumatic stress disorder
ACEs: Adverse childhood events
RAD: Reactive attachment disorder
MDD: Major depressive disorder
EB dysregulation: Emotional and behavioural dysregulation
ADHD: Attention deficit hyperactivity disorder
ID: Intellectual disability
GDD: Global developmental delay
ODD: Oppositional defiant disorder
ASD: Autism spectrum disorder
ABI: Acquired brain injury
SLI: Specific language impairment
